# Exoresection and Endoresection for Uveal Melanoma

**DOI:** 10.4103/0974-9233.65494

**Published:** 2010

**Authors:** Kaan Gündüz, Nikolaos E. Bechrakis

**Affiliations:** Department of Ophthalmology, Ankara University Faculty of Medicine, Ankara, Turkey; 1 Department of Ophthalmology, Innsbruck Medical University, Innsbruck, Austria

**Keywords:** Endoresection, Exoresection, Nevus, Melanoma, Partial Lamellar Sclerouvectomy

## Abstract

Surgical resection of uveal melanomas is an alternative eye-salvaging approach to the more commonly used irradiation techniques. There are two surgical resection techniques: Transscleral resection or “Exoresection” via a partial lamellar sclerouvectomy and “Endoresection” via a pars plana vitrectomy. While exoresection is more applicable to anteriorly located tumors with ciliary body and/or iris involvement, endoresection is more suitable for posteriorly located tumor without ciliary body involvement. Both approaches are suitable for large tumors with >8 mm in thickness. In general, eyes containing these large tumors have a very dismal prognosis regarding long-term visual function, eye retention, and irradiation-induced side effects. By removing the tumor burden from the eye, histopathologic and cytogenetic information of the tumor is available and complications associated with the so-called toxic tumor syndrome are avoided. However, both types of surgical resection are challenging surgical procedures, bearing the risk of early and late postoperative complications.

## INTRODUCTION

In many countries, the treatment of uveal melanomas is performed by various radiotherapeutical modalities using different types of ionizing radiation. In Europe, teletherapy by proton beam irradiation or brachytherapy using ruthenium-106 plaque brachytherapy is most commonly used. In the United States, besides proton-beam teletherapy, iodine-125 plaque brachytherapy is most commonly used. Parallel to the development of treatment modalities for choroidal melanomas utilizing ionizing radiation either by brachy- or teletherapy, eye retaining surgical excision of these tumors was developed either by means of “exo or transscleral resection” or by means of pars plana vitrectomy described as “endoresection.” Each of the treatment options listed has advantages and disadvantages, and thus optimal therapy is still controversial. In particular, the treatment of large uveal melanomas is a challenge because of the frequent radiogenic side effects of both brachy- and teletherapy.

## INDICATIONS

**Figure 1:**
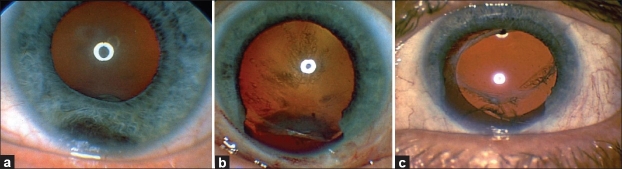
(a) Inferiorly located iridociliary melanoma. (b) After exoresection via PLSU and complete tumor removal, notching of the lens induced by the tumor and posterior subcapsular cataract are seen. (c) After phacoemulsification and intraocular lens placement, the patient achieved 20/30 vision in this eye (Reproduced from Kurt and Gündüz^11^)

**Figure 2:**
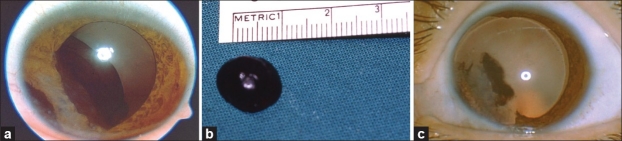
(a) Massive iridociliochoroidal melanoma producing corneal touch. (b) Gross photograph of the excised tumor showing a greater than 10-mm diameter uveal melanoma. (c) Anterior segment view 1 month after PLSU showing the absence of the iris from 7 to 11 o’clock after pupillary dilation. The corneal pigment was left untreated. (Reproduced from Kurt and Gündüz^11^)

The indications for surgical removal of uveal tumors are numerous. Removal is indicated for iridociliary tumors of indeterminate pathology that present suspicious features, such as localized pigment seeding, prominent vascularity, and increased thickness and size [Figure 1a–c].^1^,^2^ It may not be possible to perform fine-needle aspiration biopsy (FNAB) of these tumors, especially if they are located in the iridocorneal angle. Moreover, different portions of the tumor may harbor different pathologies, so small FNAB samples may be misleading. Second, iridociliary tumors with documented rapid growth, such as leiomyoma and melanocytoma, need to be surgically excised to restore intraocular structure and prevent complications such as lens subluxation and cataract.^3^ Growing necrotic iridociliary melanocytomas can cause increase in intraocular pressure (IOP); thus, removal is an option and sometimes necessary to achieve control of IOP and intraocular inflammation.^4^ Third, in many countries, plaque radiotherapy or proton beam radiotherapy for iridociliary melanomas may not be available. Plaque radiotherapy may require custom-designed plaques,^5^,^6^ and proton beam radiotherapy requires costly complex instrumentation and logistics. Both treatments may be prohibitively expensive, if they are available at all. Surgical resection may provide an alternative means of tumor control for iris and certain iridociliary melanomas under these circumstances. Fourth, thick ciliary body and choroidal melanomas require high radiation doses to the tumor and surrounding tissues, resulting in potential radiation complications. Surgical resection is believed to be safer under these circumstances [Figure 2a–c].^7^

It is generally believed that iridociliary tumors occupying <3 hours of pars plicata and choroidal melanomas <15 mm in base diameter are eligible for surgical exoresection.^8^–^10^

Endoresection of choroidal melanomas is an alternative surgical resection technique where the tumor is excised by a vitreous cutter during pars plana vitrectomy providing access to tumors located posteriorly near the optic disc and/or macula. A potential problem with this technique is the intraoperative dissemination of vital tumor cells that can lead to recurrences. Aiming to reduce the incidence of tumor cell dissemination during endoresection, a combined approach has been developed, including preoperative irradiation of the tumor with subsequent endoresection. In endoresection, there is no limitation with regard to tumor thickness, but with the largest tumor base exceeding 15 mm the intra- and postoperative complications increase. In addition, endoresection should be considered for tumors that do not infiltrate the ciliary body (due to the difficulty of visualizing this area during vitrectomy) and ideally there should be at least one disc diameter distance from the posterior tumor margin to the optic disc and fovea.

Surgical resection by either exo- or endoresection provides histopathologic and cytogenetic information of the tumor, which helps in prognostic predictions.

## EXORESECTION

### Surgical technique

The surgical technique has previously been described.^7^,^8^ Hypotensive general anesthesia is used to keep the systemic blood pressure low. During hypotensive anesthesia, it is advised to monitor brain function by registering somatic evoked potentials, to avoid brain underperfusion during the hypotensive period. A slightly reverse Trendelenburg position is preferred to reduce bleeding. After a Williams lid speculum was inserted, a 270° limbal peritomy is done centered around the tumor meridian. Three rectus muscles around the area of the tumor are hooked and isolated with 2/0 silk sutures. If the tumor is under one of the rectus muscles, the overlying muscle is disinserted using a double-armed 6/0 vicryl suture. Using blunt dissection with Steven’s scissors, the conjunctiva and Tenon’s fascia are separated away from the globe in the quadrant of the tumor. Using bipolar diathermy, episcleral blood vessels are cauterized until the bare sclera is exposed.

After the eye is rotated to the side opposite the tumor, the margins of the tumor are localized by transillumination using a bright focal light on the side of the eye exactly opposite the tumor. The margins of the tumor on the sclera are marked with a marking pencil around the tumor shadow. Subsequently, the margins of the anteriorly hinged scleral flap positioned 3–4 mm outside this mark are marked on the sclera. The shape of the scleral flap is usually rectangular to allow easier scleral suturing after the cyclectomy. With a #57 beaver blade, a groove is created to an approximately 80–90% thickness of the sclera, and the scleral flap is then developed and carried anteriorly to the corneoscleral limbus. If the blue color of the underlying choroid is not visible, the plane of dissection is gently deepened until it could be seen. Any bleeding from emissary vessels is controlled with bipolar cautery. If the tumor is in the iridocorneal angle, its shadow overlaps with the shadow of the pars plicata. In this case, the posterior margin of the tumor is marked at the posterior boundary of the pars plicata and lateral margins are marked according to the projected trajectory based on the contour of the iris tumor.

When the scleral flap is fully developed, transillumination is repeated. If the outline of the tumor can be seen after dissection of the outer lamella, this step can be skipped. A small circumferential incision using a 15° microsurgical knife is then made on the inner scleral fibers about 3 mm outside the margins of the tumor. Then, Vannas scissors are used to complete the scleral incision until the uveal tract is exposed. The uveal tract is cauterized using bipolar cautery until blanching and a gray color change of the uveal tissue is achieved.

In uveal tumors involving the iris, a 15° microsurgical knife is used to create a paracentesis port in the limbus approximately 90° from the area of the main incision, and viscoelastic is injected into the anterior chamber. The anterior chamber is then entered using a 15° microsurgical knife at the center of the flap area, and the corneoscleral incision is carried laterally with Westcott scissors. Care should be taken to avoid cutting through any tumor extending onto the corneal endothelium. For uveal tumors not involving the iris, the anterior chamber is not entered. In most cases of ciliary body and choroidal melanomas, a limited pars plana vitrectomy (20- or 23-gauge) without infusion or vitreous aspiration with a 22G needle is advised to reduce the possiblity of uveal-, retinal, and vitreous prolapse during the excision procedure. Subsequently, the uveal tumor is excised using blunt Vannas scissors. The choroidal/ciliary part of the tumor is excised first, followed by excision of the iris part if the tumor has an iris component. The pupillary rim is spared, if possible. Care is taken to leave the underlying retina or, in the case of ciliary tumors, the nonpigmented ciliary pigment epithelium intact. The uveal tumor together with the overlying scleral fibers is carefully lifted off the surgical field using a “no-touch technique” to avoid spilling tumor cells into the surgical field. If vitreous loss occurs, vitreous is removed with a vitrectomy instrument. The scleral flap is sutured to its original position with 9-0 nylon sutures. Balanced salt solution is injected into the eye via the limbal paracentesis port to adjust the IOP, if necessary. Cellulose sponges are used to check to see if the wound was watertight. If there is any leak, more 9-0 nylon sutures are placed. Any previously detached rectus muscle is sutured to its original position/insertion, and the conjunctiva is reapproximated to the limbus with 7/0 vicryl sutures.

Adjunctive plaque radiotherapy with a 20 or 25-mm round ruthenium-106 plaque is done 1 month after exoresection in uveal melanoma cases with predominant ciliary body involvement. Plaque radiotherapy is not usually done in iridociliary or iridocorneal melanomas, where the epicenter of the tumor is in the iris with limited involvement of the adjacent ciliary body. An apex dose of 100 Gy to a 1-mm depth from the inner surface of the sclera is given during plaque radiotherapy.

### Complications

Certain inherent difficulties of exoresection via partial lamellar sclerouvectomy (PLSU) can result in intraoperative, early (occurring <8 weeks after surgery), and late complications (>8 weeks after surgery). One of the major intraoperative problems is the failure to dissect a scleral flap of desired thickness.^11^ It is important to find the right plane of dissection at the time the groove is made in the sclera. If the flap is not of the desired thickness initially, as judged by the lack of the underlying blue color of uvea, the plane of dissection can gently be deepened. Care should be taken to avoid scleral perforation when dissecting or deepening the flap, as this may significantly complicate the surgery, in terms of extrascleral tumor dissemination or postoperative wound leakage.

The second major intraoperative challenge is to open the choroidal slit and dissect the uveal tissue without damaging the underlying retina or the nonpigmented ciliary epithelium.^11^ This is done most atraumatically by blunt dissection of the adjacent normal choroid with two colibri forceps. Once a slit opening is made, the surgeon should insert one blade of blunt Vannas scissors slowly into the uveal slit, taking care to stay almost parallel to the plane of dissection. Any vertical movement of the blade may cause perforation of the retina or nonpigmented ciliary epithelium and vitreous loss. We have found that vitreous loss and subsequent vitrectomy do not lead to retinal detachment, as long as the tumor base remains anterior to the muscle insertions. We first remove the prolapsed vitreous using the vitrectomy cutter away from the wound margins, then close the scleral flap with three to five sutures and do vitrectomy to remove the residual vitreous at the wound margins. This technique is safer compared to an open sky vitrectomy approach because of the decreased risks of hypotony and possible retinal damage. Problems with retinal detachment may be more likely to occur in tumors located posterior to the muscle insertions.

**Figure 3:**
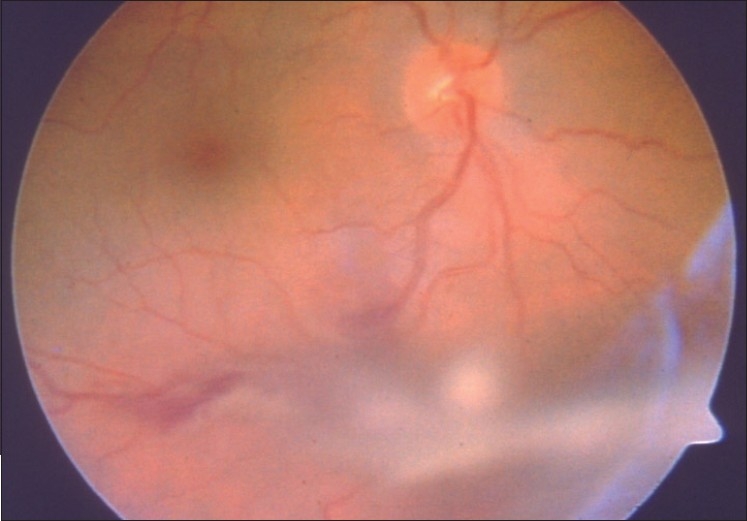
Fundus photograph showing mild vitreous hemorrhage one day after surgery in an eye that underwent PLSU

Early postoperative complications include vitreous hemorrhage [Figure 3], hyphema, subretinal hemorrhage, transient blepharoptosis, cataract, retinal detachment, choroidal detachment, corneal edema, and elevated IOP.^8^–^12^ The most common early complication after PLSU surgery is vitreous hemorrhage.^9^ Vitreous hemorrhage was reported to occur in 28 of 95 (29%) patients in the series of Shields *et al*.^9^ It generally occurs from inadequately cauterized choroidal or ciliary body blood vessels.^8^,^9^ In general, vitreous hemorrhage resolves spontaneously; however, when it persists beyond 8 weeks, pars plana vitrectomy can be performed. Persistent vitreous hemorrhage was seen in only 2 of 22 (9%) patients in the series of Kurt and Gündüz^11^ and in 3 of 95 (3%) patients in the series of Shields *et al*.^9^

**Figure 4:**
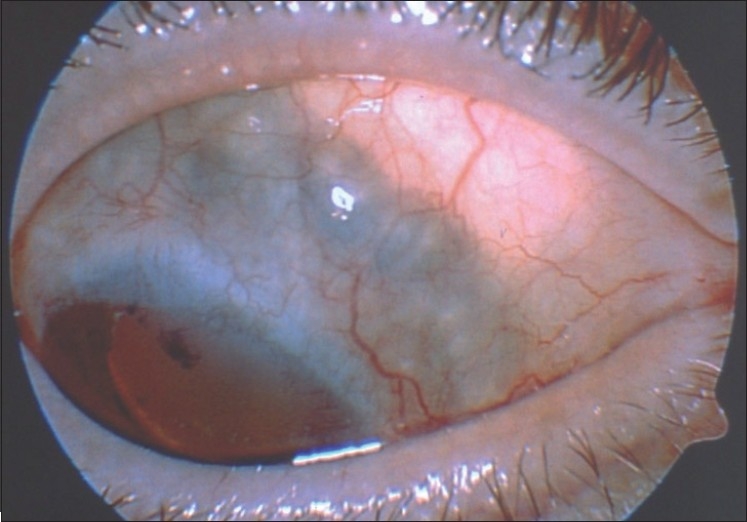
Scleral thinning in the superonasal quadrant of an eye that underwent PLSU for an iridociliary melanoma (Reproduced from Kurt and Gündüz^11^)

Late postoperative complications include cataract, subretinal fibrosis, posterior synechiae, rhegmatogenous retinal detachment with or without proliferative vitreoretinopathy, cystoid macular edema, elevated IOP, scleral thinning, and rarely iris neovascularization.^8^–^12^ Iatrogenic lens damage will lead to cataract formation during or after surgery. However, often, there is a slow and progressive cataract development many months after surgery, occurring as a late complication. It is believed that if the retrolenticular vitreous is not disturbed, the risk of cataract decreases.^8^ We have found that it is important to use a paracentesis port rather than the main corneoscleral incision for purposes of injecting viscoelastic into the anterior chamber, anterior chamber wash-out, or inflating the eye at the end of surgery. This approach poses less risk of inadvertently damaging the lens. Eleven of 22 patients (50%) required cataract surgery with phacoemulsification and IOL implantation in the series of Kurt and Gündüz.^11^ After PLSU, zonular defects can occur, and phacoemulsification surgery should be performed with care. Cases with more than 3 clock hours of zonular defects are more likely to be complicated; cataract surgery in these cases should be performed by an experienced phaco surgeon.^13^ Scleral thinning can also be a worrisome problem, probably indicating a flap thickness problem [Figure 4].^11^ Usually, this problem can be followed with observation unless marked bulging of the uveal tract develops, in which case scleral reinforcement with a patch graft should be considered.

In a comparative analysis of eyes having large uveal melanomas (thickness >7 mm) treated with I-125 brachytherapy versus PLSU, Bechrakis *et al*. found that the incidence of secondary glaucoma was significantly higher after I-125 brachytherapy (33.3%) than after PLSU surgery (5.6%) (*P* = 0.03). No difference was found with respect to eye retention (*P* = 0.3). Their study suggests that eyes with large uveal melanomas, which are eligible for transscleral resection have a better long-term visual function and lower incidence of radiation-induced complications such as neovasularization and secondary glaucoma, when compared to eyes treated by I-125 brachytherapy.^14^

### Tumor recurrence

Tumor recurrence may follow incomplete removal of malignant tumors with positive surgical margins. To avoid this serious problem, adjunctive plaque radiotherapy has been suggested. In the series of Damato *et al*. and Bechrakis *et al*., adjunctive plaque radiotherapy was shown to be effective for decreasing local tumor recurrence after PLSU.^15^,^16^ Histopathologic study of enucleated eyes harboring melanoma show melanoma cells in the sclera in 56% of cases.^17^ The presence of untreated intrascleral melanoma cells is another reason to perform plaque radiotherapy after PLSU.^15^ Kim *et al*. speculated that dissemination of melanoma cells during PLSU may account for occurrence of noncontiguous new tumor after surgery.^18^ Plaque radiotherapy using Ru-106 plaques is usually performed in patients with ciliary, ciliochoroidal, and iridociliochoroidal melanoma but not in cases where the epicenter of the tumor is in the iris with little involvement of the ciliary body. Bechrakis *et al*. reported that in addition to lack of adjuvant radiotherapy, larger basal tumor diameter, older age, and preoperative retinal detachment increased the incidence of tumor recurrence.^16^

### Distant metastasis

Distant metastasis presents a challenging problem in patients with uveal melanoma. In the series of Damato *et al*., predictors for metastatic disease after PLSU included: epithelioid/mixed cell type; largest basal tumor diameter ≥16 mm; patient age greater than 60 years; secondary enucleation for gross residual or recurrent tumor; secondary enucleation for extraocular extension; and superior tumor location.^19^ Memmen and McLean found that epithelioid/mixed tumor cell type and involvement of the surgical margin by the melanoma were risk factors for metastasis after surgical resection.^20^ Bechrakis *et al*. found that the 5- and 10-year metastatic rates were 28% and 44% and were significantly affected from extraocular spread, tumor thickness, and local tumor recurrence.^16^ In the series of Shields *et al*., 5 of 95 patients (7%) developed metastatic disease, whereas in the series of Damato *et al*., 52 of 332 patients (15%) had metastatic disease.^9^,^19^ Bechrakis *et al*. and Augsburger *et al*. found no statistically significant difference in the survival rate of patients treated with PLSU versus those treated with plaque radiotherapy.^14^,^21^

### Visual outcomes

Visual outcomes appear to be better after PLSU than after radiation treatment.^14^,^21^,^22^ Bechrakis *et al*. found that 61.1% of eyes harboring large uveal melanomas retain a visual acuity of ≥20/200 after PLSU versus 5.6% of eyes after I-125 brachytherapy, the difference being statistically significant (*P* < 0.0009).^14^

## ENDORESECTION

### Surgical technique, complications, and prognosis

In phakic patients, it is advisable to perform microincisional phacoemulsification with IOL implantation before starting pars plana vitrectomy in order to get better access to the vitreous base during the vitrectomy procedure. Following phacoemulsification, a conventional (20-gauge) 3-port pars plana vitrectomy is performed by inducing a complete posterior vitreous detachment by suction with the vitreous cutter, care being taken to also detach the posterior hyaloid from the anterior tumor margin. It is advisable to use a fourth port for an additional endoilluminating device such as chandelier endoillumination to facilitate bimanual manipulations during endoresection. After endodiathermy of the retinal vessels over the tumor base, the subretinal fluid is drained through a peripheral retinectomy after application of 1–2 cc perfluorcarbon liquid to the posterior pole. This is followed by transretinal endoresection with increased IOP (up to 85 mmHg) and low systolic blood pressure (≤90 mmHg) to minimize bleeding from tumor vessels. The tumor is excised up to the sclera accompanied by endodiathermy and/or endolaser of individual scleral ciliary arteries and choroidal vessels. After flattening the retina by further perfluocarbon injection, the retinectomy and choroidectomy edges are coagulated by an endolaser in continuous-wave mode and by cryocoagulation for the anterior endoresection and retinectomy borders. Flat tumor residues still remaining at the bare sclera are subjected to hyperthermia using 810-nm diode endolaser with low energy and continuous wave settings. Perfluorcarbon liquid is then directly exchanged for 5000 centistokes (cSt) silicone.

Internal eyewall resection or endoresection has been proposed as an alternative to irradiation by Peyman *et al*. and Damato *et al*. in the past.^23^–^28^ A potential problem with the technique hitherto described by these authors is the intraoperative dissemination of vital tumor cells that can lead to recurrences.^29^–^31^ There is considerable controversy on the issue of the need of preoperative “neoadjuvant” irradiation before endoresection. Bechrakis *et al*. has proposed preoperative irradiation in order to “devitalize” the tumor and perform endoresection a few days postirradiation.^32^–^34^ The most adequate form of preoperative irradiation is teletherapy by proton beam treatment due to its uniform dose distribution and maximum spring of critical structures such as the fovea and/or the optic disc. As an alternative to proton beam, gamma-knife radiosurgery or other forms of highly conformal external beam radiotherapy have been proposed.^35^ Unfortunately, there are no prospective comparative data available addressing this issue, but there is considerable concern by numerous specialists in this field, that endoresecion without prior irradiation to the tumor, that is tumor “devitalization,” will increase the recurrence and metastasis rates. The reasoning of tumor resection after tumoricidal irradiation to the tumor can be described as addressing the issue of the so-called “toxic tumor syndrome,” namely the removal of already necrotic or potentially necrotic tumor tissue that produces or is expected to produce in the future hypoxia and necrosis induced cytokines, inducing further radiation-related retinal and optic nerve toxiciy. By removing or debulking the irradiated tumor and reattaching the detached retina, long-term ocular morbidity could be reduced significantly, especially in large uveal melanomas that have undergone irradiation.^32^

**Figure 5:**
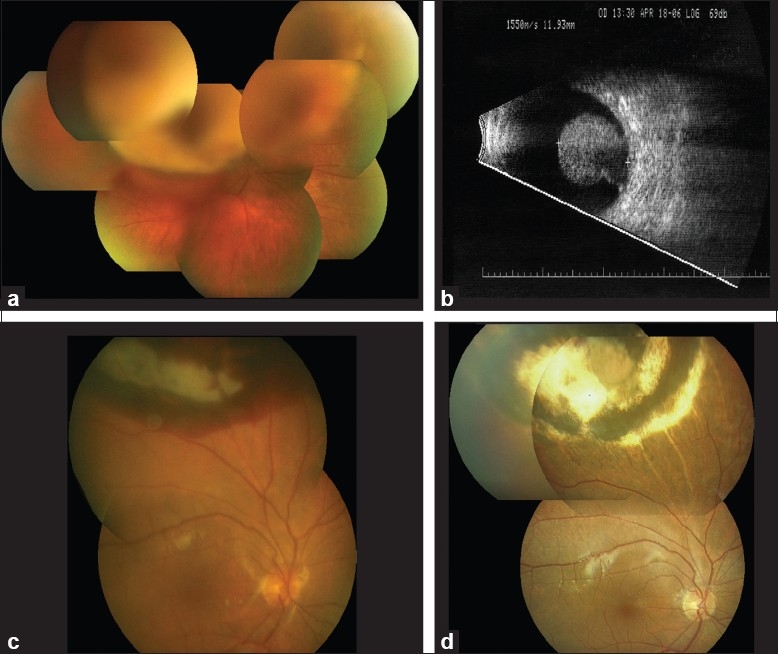
(a) Large choroidal melanoma obscuring funduscopic view to the posterior pole. Visual acuity is 20/100. (b) B-scan ultrasound of the tumor depicted in Figure 4(a) showing a 11.9-mm-thick mushroom-shaped choroidal melanoma. (c) Early postoperative view of the fundus of the eye depicted in (a) 2 days after endoresection. Visual acuity is 20/200. (d) Late postoperative view of the fundus of the eye depicted in (a) 18 months after endoresection. Visual acuity is 20/25.

Endoresection after preoperative neoadjuvant proton beam irradiation is a very promising procedure for treating highly prominent choroidal melanomas located near the posterior pole in close proximity to the optic nerve or macula [Figure 5a–d] These posteriorly located tumors can only be managed by transscleral resection with increased surgical risks.^32^ Brachytherapy and teletherapy methods are also associated with increased vision and globe loss risks in large uveal melanomas. Shields *et al*. found that 68% of patients with a large melanoma having tumor thickness >8 mm have poor visual acuity (20/200 to no light perception vision) after plaque radiotherapy at 10 years follow-up.^36^ In another study, Shields *et al*. reported that 66% of the eyes with large uveal melanomas retained their eyes and the mortality was 55% at 10 years.^37^ Conway *et al*. reported that following proton beam radiotherapy, 75% of the patients with large melanomas >10 mm in thickness had visual acuity <20/200 at a median follow-up of 28 months. The globe retention rate was 67% at this relatively short term.^38^

Long-term observations will have to demonstrate whether this newly developed combined radiotherapy plus –endoresection surgery concept can effectively prevent serious radiation-induced eye-endangering side effects such as those associated with radiation monotherapy of large melanomas. However, with a mean follow-up of 2 years these high-risk patients—in which enucleation would have been the alternative treatment option—did not have increased morbidity compared to patients with low-risk choroidal melanomas regarding treatment-related ocular side effects.^33^

## CONCLUSIONS

Although surgical intraocular tumor resection techniques pose some difficulties and are technically challenging, they are effective alternative treatment options in selected cases of uveal tumors. Exoresection should be reserved for anteriorly located tumors with ciliary body infiltration. In tumors extending posterior to the ora serrata into the choroid, separation of the retina from the tumor can be difficult and is more frequently associated with intraoperative complications the closer, the posterior tumor margin is located to posterior pole. In cases of choroidal melanoma without any ciliary body infiltration, endoresection with preoperative irradiation is more advisable since intraocular bleeding can be more effectively monitored and controlled intraoperatively. Moreover, intraocular and extraocular vital tumor seeding is prevented effectively by preoperative “neoadjuvant” irradiation. In general, intraocular tumor resection surgery can be associated with postoperative complications requiring frequent patient monitoring. However, in the long run local tumor resection either by means of exoresection (transscleral resection) or by means of endoresection can be advantegeous to primary irradiation modalities, that can cause significant radiation-induced side effects, especially in the case of large tumors.
